# Secondary Metabolites from *Inula britannica* L. and Their Biological Activities

**DOI:** 10.3390/molecules15031562

**Published:** 2010-03-10

**Authors:** Abdul Latif Khan, Javid Hussain, Muhammad Hamayun, Syed Abdullah Gilani, Shabir Ahmad, Gauhar Rehman, Yoon-Ha Kim, Sang-Mo Kang, In-Jung Lee

**Affiliations:** 1 School of Applied Biosciences, Kyungpook National University, Korea; 2 Department of Chemistry, Kohat University of Science & Technology, Kohat, Pakistan; 3 Department of Genetic Engineering, School of Life Sciences & Biotechnology, Kyungpook National University, Korea; 4 Department of Biotechnology, Kohat University of Science & Technology, Kohat, Pakistan

**Keywords:** *I. britannica*, sesquiterpenoids, flavonoids, triterpenoids, anticancer, antioxidant, anti-inflammatory, hepatoprotective, neuroprotective

## Abstract

*Inula britannica* L., family *Asteraceae*, is used in traditional Chinese and Kampo Medicines for various diseases. Flowers or the aerial parts are a rich source of secondary metabolites. These consist mainly of terpenoids (sesquiterpene lactones and dimmers, diterpenes and triterpenoids) and flavonoids. The isolated compounds have shown diverse biological activities: anticancer, antioxidant, anti-inflammatory, neuroprotective and hepatoprotective activities. This review provides information on isolated bioactive phytochemicals and pharmacological potentials of *I. britannica*.

## 1. Introduction

**Figure 1:**
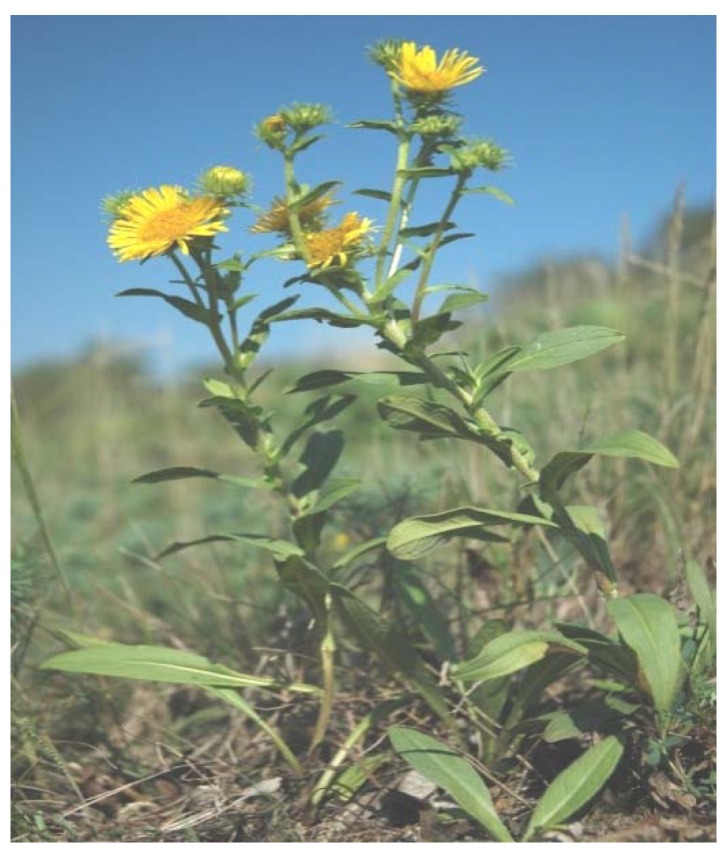
*Inula britannica* (courtesy Mr. Ivan Bilek, Nature photo).

The genus *Inula* (Asteraceae) is known for diverse biological activities, *i.e.*, anticancer, antibacterial, hepaprotective, cytotoxic, and anti-inflammatory properties [1]. It comprises about 100 species distributed in Asia, Europe and Africa [2]. *Inula britannica* (Figure 1) is an important plant species used in Traditional Chinese Medicine (TCM) and Kampo Medicines. Along with *Inula japonica*, it is known as ‘Xuan Fu Hua’ in TCM. It is also known as British yellowhead or meadow fleabane.

*I. britannica* is an erect biennial or perennial plant and is species of eutropic and disturbed grassland [3]. It is 15 to 75 cm tall and its stem is covered with oppressed hairs or without hairs. The flowers of the plant are bright yellow, standing alone or in clusters of 2–3 [4]. A common configuration of the plant is to have a mother plant surrounded by 8 to 10 satellite plants connected by rhizomes [2]. The plant prefers sandy loamy and clayey soil [5].

More than 80% of the marginal communities rely, one way or the other, on various medicinal plants for curing diseases using traditional knowledge [6]. *I. britannica*, in combination with other plants, is used for nausea, hiccups and excessive sputum [7]. Its flowers are used for treatment of intestinal diseases, bronchitis and inflammation [8,9,10,11]. In TCM, a decoction of aerial parts or flowers is used for asthma, and as an expectorant [12,13,14]. The flowers are used as antibacterial, carminative, diuretic, laxative, stomachic, tonic rapid-healer, for hepatitis and tumors [9,13,14]. Being such an important medicinal plant, scientists have examined various aspects of the plant. In the present review, we focus on the biologically active secondary metabolites and their potential pharmacological roles.

### 1.1. Perspective Secondary Metabolites

Various chemical constituents have been isolated from *I. britannica* and reported (Table 1). These include steroids, terpenoids (sesquiterpene lactones, diterpenes and triterpenoids), phenolics and flavonoids. The literature shows that almost 102 compounds have been isolated and purified from this plant [15]. Most of the reported compounds are from the flowers or aerial parts of the plant. Due to the richness of its chemical constituents in aerial parts or flowers, the roots have been largely neglected so far. In the present review, only bioactive metabolites purified from *I. britannica* and their biological potential are discussed. Major bioactive secondary metabolites and their biological effect have been summarized in Table 2.

**Table 1:** Reported chemical constituents from *I. britannica.*

Chemical compound(s)	Part/Fraction	Reference(s)
Bisdesacetylbritannin; dihydrodihydrobritannin; acetyldihydrobritannin; bisdesacetyldihydrobritannin; methyl ester of 2α,6α-diacetoxy-4β-hydroxy-7α(*H*),8,10β(*H*)-pseudoguai-8,12-olidylmethylenethioacetic acid and methyl ester of 2α,6α-diacetoxy-4β-hydroxy-7α(*H*),8,10β(*H*)-13-methylpseudoguai-8,12-olidylmethylenethioacetic acid	Derivatives/synthesis	[16, 17, 18, 19]
2-*O*-Alkyloxime-3-phenyl)-propionyl-1-*O*-acetylbritannilactone esters	Derivatives/Synthesis	[20]
Britannilide, oxobritannilactone, eremobritanilin	Flowers /Ethyl acetate	[21, 22]
Pulchellin C	Flowers/Acetone	[23]
Inuchinenolides A, B, and C, tomentosin, ivalin, 4-epi-isoinuviscolide, gaillardin	Aerial / Ethyl acetate	[24]
4α,5β-Epoxyeupatolide; 4α,5β-epoxydesacetylovatifolin; 5α-hydroxydehydroleucodin; 14-hydroxy-2-oxoguaia-1(10),3-dien-5α,11βH-12,6α-olide and 2-oxo-8α,10β,dihydroxyguai-3-en-1-α,6β,11β*H*-12,6-olide	Flowers	[26]
Salicylic, *p*-hydroxybenzoic, protacatechuic, vanillic, syringic, p-hydroxyphenylacetic, *p*-coumaric, caffeic, and ferulic acids	Aerial parts	[28]
2,3,4,5-Tetrahydro-1-benzooxepin-3-ol,	Essential oils	[29]
Kaurane glycosides- Inulosides A and B	Flowers/Butanol	[30, 72]
Triterpene fatty acid esters, 3β,16β–dihydroxylupeol 3-palmitate 3β,16β-dihydroxylupeol 3-myristate, 6-hydroxykaempferol 3-sulfate; epi-friedelinol, β -amyrin palmitate, olean-13(18)-en 3-acetate, sitosteryl 3-glucoside; quercetin 3-sulfate	Aerial parts	[31]
Britanlins A, B, C, D	Dried flower/Ethanol extract	[75]

**Table 2:** Summary of biologically active compounds from *I. britannica.*

Compound	Plant part	Extract/Fraction	Yield	Activity	Standard	Ref
1-*O*-Acetylbritannilactone (**2**)	Flower	95% EtOH	1.1 g	Cytotoxic, apopotic, inflammation	Streptomycin	[3,25,57,58]
1,6-*O,O*-diacetylbritannilactone (**3**)			32 mg			
6*α*-*O*-(2-methylbutyryl)-britannilactone (**11**)			63 mg			
Neobritannilactone A (**9**)			15 mg			
Neobritannilactone B (**10**)			102 mg			
Inulanolides A (**5**)	Aerial part	EtOAc	9 mg	Inflammation	Nm	[37]
Inulanolides B (**6**)			31 mg			
Inulanolides C (**7**)			89 mg			
Inulanolides D (**8**)			37 mg			
Ergolide (**4**)	Flowers	80% MeOH	110 mg	Inflammation iNOS, NF-_K_B, I_K_B, COX-2	Nm	[35]
Taraxasteryl acetate (**13**)	Aerial part	CHCl_3_	39 mg	Hepato--protective	Nm	[38]
Patuletin (**14**)	Flowers	80% MeOH	70 mg	Antioxidant,	Garlic acid/DPPH	[4,39]
Axillarin (**18**)			60 mg			
Nepitrin (**21**)			60 mg			
Quercetin (**27**)	Flowers	95% EtOH	1.2 g	Antioxidant, balloon injury, cytotoxic	DPPH	[43,73]
Spinacetin (**28**)			75 mg			
Diosmetin (**24**)			32 mg			

Nm = Not mentioned.

#### 1.1.1. Sesquiterpenes

**Figure 2:**
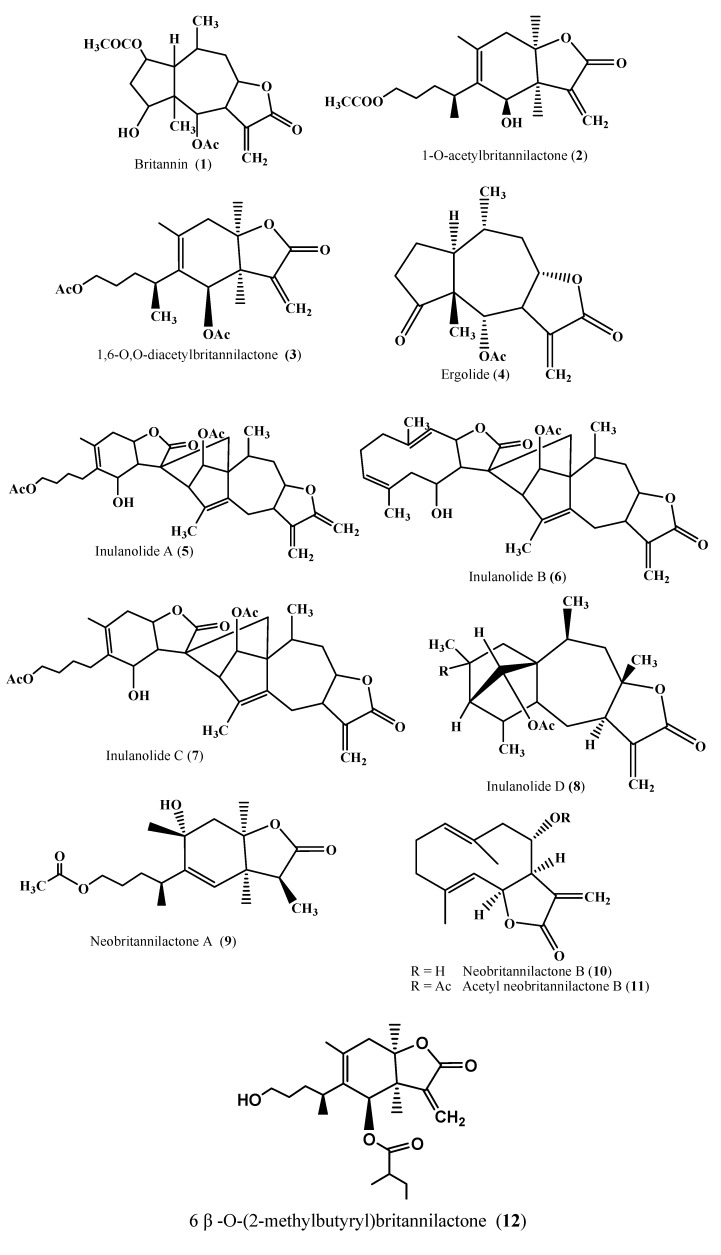
Bioactive metabolites isolated from *I. britannica.*

Britannin (**1**) was the first compound purified from the ethanolic fraction of aerial parts of *I. britannica.* It led to the isolation and synthesis of physiologically active compounds [16] (Table 1). Among sesquiterpenes, the most significant are 1-*O*-acetylbritannilactone (OABL) (**2**) and 1,6-*O,O*-diacetylbritannilactone (OODABL) (**3**). These were purified from the chloroform fraction of flowers using advanced chromatographic and spectroscopic techniques [25,32]. These have been reported to work against oxidative stress and are cytotoxic [33]. OODABL has one α,β unsaturated sesquiterpene lactone and two acetyl moieties, while OABL has one acetyl group [34]. Acetyl groups at positions 1 and 6 in OODABL have been found to be more cytotoxic in human leukemia cell-60 compared to OABL [34] (Figure 2).

Cytotoxicity-guided isolation resulted in identification of 4α,6α-dihydroxyeudesman-8 β,12-olide, ergolide, 8-epi-helenalin, and bigelovin from the flowers of *I. britannica* [35]. The presence of exo-methylene group in ergolide has made it more reactive thus presenting higher biological activity. Ergolide (**4)** inhibits inducible nitric oxide synthase and cyclo-oxygenase-2 expression in RAW 264.7 macrophages through inactivation of NF-_K_B [36] (Figure 2).

A bioassay guided isolation from ethyl acetate fraction of aerial parts of *I. britannica* resulted in exploration of four new sesquiterpene dimmers bearing a norbornene moiety, inulanolide A (**5**) B (**6**) C (**7**) and D (**8**) and three known sesquiterpenes [37,80]. Inulanolide B and D have exhibited potent inhibitory effect on the LPS-induced NF-_K_B activation [37].

From air dried powdered flowers of *I. britannica,* three new sesquiterpenes - neobritannilactone A (**9**), neobritannilactone B (**10**), acetyl neobritannilactone B (**11**) and 6β-*O*-(2-methylbutyryl)britannilactone (**12**) along with known compounds *i.e.,* britannilactone, 1-*O*-acetylbritannilactone (**2**) and 1, 6-*O,O*-diacetylbritannilactone (**3**) - were obtained and identified using 1D, and 2D NMR [3,25].

#### 1.1.2. Triterpenoids

**Figure 3:**
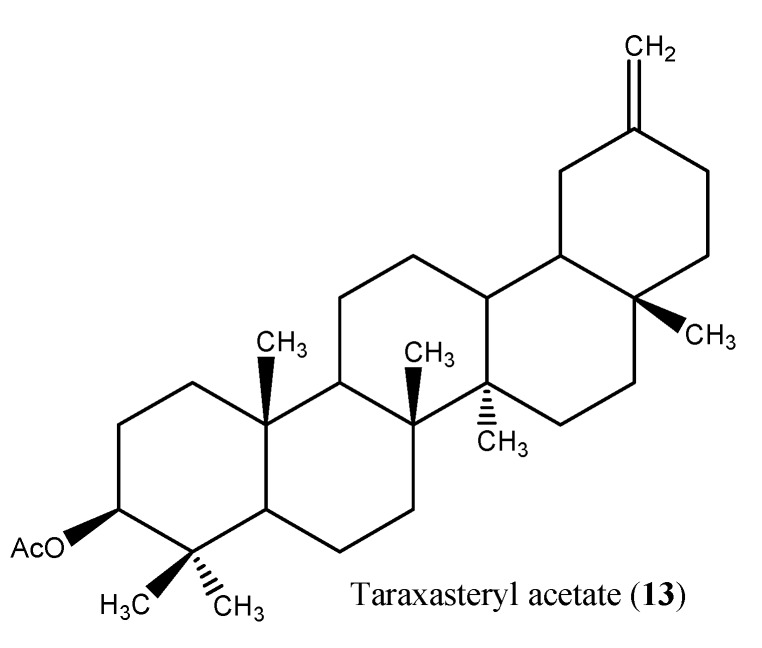
Bioactive terpenoids from *I. britannica*.

Three triterpenoids [taraxasteryl acetate (**13**), β-amyrin and lupeol] and three steroids (β-resasterol and stigmasterol and ψ-taraxasterol) were purified from the chloroform fraction of the aerial parts of the plant [27] (Figure 3). Besides that, two new triterpenoids fatty acid esters, 3β,16β dihydroxylupeol 3-palmitate and 3β,16β-dihydroxylupeol-3-myristate were also isolated [31]. Taraxasteryl acetate has been found very effective in preventing activity against acute hepatic failure induced by PA and LPS in a concentration dependent manner [38].

**Figure 4:**
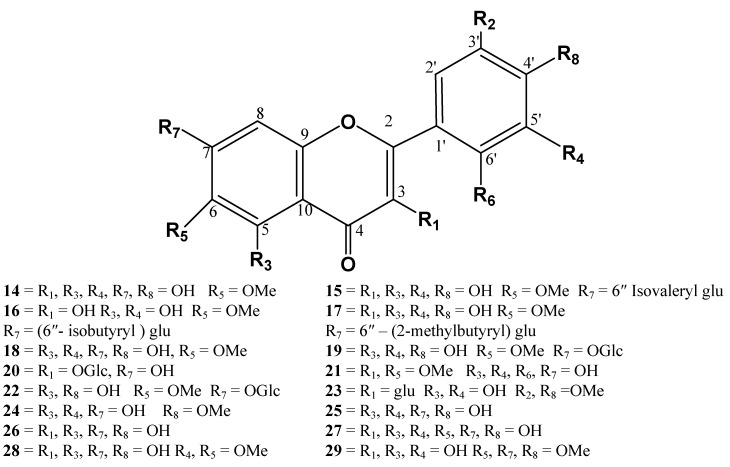
Flavonoids from *I. britannica*.

#### 1.1.3. Flavonoids

Flavonoids were isolated from the butanol fraction of *I. britannica* (Figure 4). These include patuletin (**14**), luteolin, patulitrin, patuletin 7-*O*-(6''-isovaleryl)glucoside (**15**), patuletin 7-*O*-(6''-isobutyryl) glucoside (**16**), patuletin 7-*O*- [6''-(2-methylbutyryl)] glucoside (**17**), nepetin (**18**), nepitrin (**19**), kaempferol 3- glucoside (**20**), axillarin (**21**), hispiduline-7-glucoside (**22**), and isorhamnetin 3-glucoside (**23**) [4,39]. From butanol fraction of the flowers, four flavonoids: 4,5,7-trihydroxy-3,6-dimethylflavone-7-*O*-β-D-glucopyranoside, isohamnetin-3-*O*-β-D-glucopyranoside, rhamnetin-3-*O*-β-D-glucopyranoside and kaemferol-3-*O*-β-D-glucopyranoside were identified [30]. These flavonoids play a vital role in enzyme inhibition, are antioxidant and posses cytotoxic activities [40,41,42].

In continuation to the exploration of chemical constituents from its aerial parts, eight more flavonones were identified: (i) luteolin; (ii) diosmetin (**24**)**;** (iii) chrysoeriol (**25**)**;** (iv) kaempferol (**26**)**;** (v) quercetin (**27**); (vi) 6-hydroxyluteolin-6-meether; (vii) spinacetin (**28**)**;** and (viii) eupatin (**29**) [43].

## 2. Pharmacological Significance

### 2.1. Antioxidant Activity

There is a delicate balance between generation and destruction of oxidant agents. These may be beneficial or deleterious to the organism [44,45,46]. Under physiological conditions, the level of reactive oxygen species is maintained low by the activity of antioxidative systems that include secondary plant metabolites and scavenging enzymes [45,47]. Many *in vitro* studies indicate that compounds like sesquiterpenes, flavonoids, coumarins and phenolic acids can have substantial antioxidant activity [48,49]. Flavonoids have been extensively studied for antioxidant activities [50].

As for the flavonoids, various flavonones like patuletin, luteolin, patuletin 7-*O*-(6''-isovaleryl) glucoside, patuletin 7-*O*-(6''-isobutyryl) glucoside, patuletin 7-*O*- [6''-(2-methylbutyryl)] glucoside, nepetin, nepitrin, kaempferol 3-glucoside, axillarin, hispiduline 7-glucoside, and isorhamnetin 3-glucoside were evaluated for antioxidant activity using DPPH assay [39]. According to results, the IC_50_ values of luteoline, patuletin 7-*O*-(6''-isovaleryl) glucoside and patuletin 7-*O*-(6''-isobutyryl) glucoside were 11.7, 10.6 and 11.2 µg/mL respectively while the positive control (garlic acid) had an IC_50_ value of 3.7 µg/mL [39].

Besides the aforementioned flavonoids, luteolin, diosmetin, chrysoeriol, kaempferol, quercetin, 6-hydroxyluteolin-6-methyl ether, spinacetin, and eupatin were also explored for their efficiency towards oxidative stress [43]. The antioxidant assay (using DPPH) revealed that luteolin, diosmetin, chrysoeriol, kaempferol, quercetin, 6-hydroxyluteolin-6-methyl ether, spinacetin and eupatin showed 85.6, 55.4, 53.6, 61.0, 85.4, 86.3, 85.4, and 84.9% activity, respectively [50]. Looking at findings of various studies, there is still a need to fix the pathway and overall enzymatic behavior of these molecules under biotic and abiotic stresses, both at plant and animal levels.

### 2.2. Anti-Cancer Activities

Natural products are now considered as the most important source of curing various mortal diseases. *I. britannica*, like many other plant species e.g., *Rhazya stricta* [51], *Annona coriacea*, *A. glabbra*, *etc.* [52], *Teucrium royleanum* [53] and many other plants and their products [54], has been found to display anticancerous activity. *I. britannica* is also reported to be used against tumor in TCM [15]. Looking at the indigenous uses of plant, two sesquiterpeniods, OABL (**2**) and OODABL (**3**) were isolated, which induced phosphorylation of BCl-2 (anti-apoptotic protein) in breast, ovary and prostate cancer cell line. Phosphorylation of BCl-2 was important for OODABL induced cytotoxicity [33,55,56].

OODABL has also been found inducing Bcl-2 phosphorylation in MCF-7 cells (cell lines for breast cancer) [57]. Various concentrations (1.25, 12.5, 25. 50 and 100 µM) of OODABL resulted with IC_50_ value of 12.5 µM in MCF-7 cells. Similarly OABL was also tested while using dose of 0.3 nM, 3 nM, 30 nM, 300 nM, 3 µM, and 30 µM in MCF-7 cells that showed that OABL has lesser cell viability with an IC_50_ of 200 µM. To clarify the role and function of OODABL and OABL in cytotoxicity, various cell lines (e.g., PA-1, DU-145, NCI-H-60 and NIH 3T3) were tested in different concentrations. Ho *et al.* have suggested in their patent that it can be used for prevention and treatment of cancer. Still there is a need for further clinical trials to prove the role of OODABL and OABL [57]. Besides that, mechanism of its role and function needs to be convoluted.

Some of the recent studies indicate that OODABL can also induce the occurrence of apoptosis in human leukemia cells (HLC) [58]. To know its effect in different concentrations, cells were treated with britannilactone (BL), OABL, and OODBL. Results indicated that BL had less ability of apoptosis in HL-60 cells. OABL has induced apoptosis with 50 and 100 µM, while the percentages of apoptotic cells were 20.13 and 40.2%. OODABL was more potent inducer of apoptosis in HL-60 cells with 20.04, 20.51, 49.86, 58.76, and 64.23% of apoptotic cells with 5, 10, 25, 50, and 100 lM OODABL, respectively [58]. The study also suggested that OODABL had an acetyl group in position 1 and 6 that is cytotoxic in HL-60 cells than OABL. Detailed mechanism has been reported that proves that OODABL is helpful in *in-vitro* anticancer activities [58].

Besides OABL and OODABL, other sesquiterpenes lactones isolated from *I. britannica* have also been tested for cytotoxic activities. The effects against various cell lines (COLO 205, HT 29, human AGS gastric carcinoma cell lines (CCRC 60102) were tested for 6β-*O*-(2-methylbutyryl) britannilactone, neobritannilactone A, B and acetyl neobritannilactone B. Neobritannilactone B and acetyl neobritannilactone B appeared to be more potent apoptosis-inducing agents than neobritannilactone A and 6β-*O*-(2-methylbutyryl) britannilactone for COLO 205, HT-29, AGS, and HL-60 cells. The percentages of apoptotic COLO 205, HT-29, HL-60, and AGS cells were 41.62 and 76.87%; 66.54 and 69.70%; 77.57 and 95.17%; and 11.78 and 9.89% after 24 h of incubation with neobritannilactone B and acetyl neobritannilactone B (25 µM), respectively [3].

### 2.3. Neuroprotective Activities

Neuroprotection is the term used to describe prevention or delay of pathological neuronal loss in diseases of the central nervous system [59,60]. Efforts have been made to overcome these neuronological diseases. *I. britannica* being used in TCM for such purpose motivated phytochemists and pharmacologists to know the neuroprotective potential of its chemical constituents. Neuroprotective activities were evaluated by monitoring the viability of primary cultures of rat cortical cells from oxidative stress induced by glutamate in DPPH and MTT assays. Subjecting patuletin (**14**), nepetin (**18**) and axillarin (**21**) for these assays, concentration gradient of 1, 10 and 50 µM was used. According to results, protective effect of patuletin (**14**), nepetin (**18**) and axillarin (**21**) was 51.8, 49.8 and 60.6% after pre-treatment and 70.7, 57.9 and 55.4% by post-treatment, using 50 µM concentrations on cell viability of the cultures. However, the optimal doze of control and glutamate-insulted was 1.335% and 0.938%, respectively [4].

### 2.4. Anti-Inflammatory Activities

Inflammation is regarded as a dynamic process that elicits in response to microbial infections, mechanical injuries and burns. This process involves changes in blood flow and increased vascular permeability, destruction of tissue via inflammatory mediators, such as prostaglandins (PGs) leukotriene platelet activating factors induced by phosphliphase A_2_, cyclooxygenase (COXs) and lipoxygenases [61]. Classical example for treating inflammation is sesquiterpenes lactones from *I. britannica* to inhibit NO synthesis [62]. iNOS and COX-2 expression, and NF-κB activation have been used as biomarkers for the screening of anti-inflammatory activity. OABL inhibit iNOS and COX-2, responsible for suppression of NO and PGE2 synthesis in RAW 264 macrophage [34]. A concentration of 10 μmol/L of OABL has inhibited the production of NO and PGE_2_ in LPS/IFN-γ -stimulated RAW 264.7 macrophages while adopting western blot analysis, electrophoretic mobility shift assay and MTT assay [34].

OABL has also been explored to know the effect on neointimal hyperplasia after balloon injury and its mechanism of action in rats (Sprague-Dawley). A concentration of 26 mg/kg of OABL and polyglycol (control) was used daily from 3 days before injury to 2 weeks after balloon injury. OABL showed significant reduction in neointimal formation. Activity ratio of OABL and control was 1.94 and 0.84, respectively. These findings suggest that OABL is a potential inhibitor of neointimal formation because it blocks injury-induced NF-_K_B activation, and may have beneficial effects in reducing the risk of restenosis after angioplasty [63]. Results of another study on OODABL show that acetyl moieties add to the lipophilicity, and consequently enhance cellular penetration so that OODABL possess the most anti-inflammatory effect and may be a potent lead structure for the development of therapeutic and cytokine-suppressing remedies suitable for the treatment of various inflammatory diseases [73,74]. Similarly, Inulanolides B and D exhibited potent inhibitory effect on the LPS-induced NF-_K_B activation [37]. In another study, higher doze of total flavonoid extracts (mainly quercetin, luteolin, 6-methoxyluteolin, spinacetin and isorhamnetin) of aerial parts of the plant inhibited the neointimal hyperplasia induced by balloon injury [73].

Ergolide markedly decreased the production of prostaglandin E (2) (PGE_2_) in cell-free extract of LPS/IFN-gamma-stimulated RAW 264.7 macrophages in a concentration-dependent manner, without alteration of the catalytic activity of COX-2 itself. These results demonstrate that suppression of NF-_K_B activation by ergolide might be attributed to the inhibition of nuclear translocation of NF-_K_B resulting from degradation of I_K_B and the direct modification of active NF-_K_B. This lead to suppression of the expression of iNOS and COX-2 that play important roles in inflammatory signaling pathway [64]. It has also been classified as cyclooxygenase inhibitor for COX-2 [65].

In TCM, *I. britannica* is used in asthma, as a warming expectorant and for phlegm removal. As for asthma, it is inflammatory disease of airways and flavonoids have been reported to strengthen the connective tissues by reducing histamine levels [78]. Certain flavonoids like luteoline and quercetin have been reported to play a lead role in alleviating bronchoconstriction and hyperreactivity *i.e.*, anti-asthma [77,79,80]. In case of *I. britannica,* although a little or no focus at molecular/clinical level for anti-asthma have been found but the aforementioned purified chemical constituents from the plant have been reported elsewhere for the same purpose, thus supporting the traditional use in TCM.

### 2.5. Hepatoprotective Affect

Relying on the traditional Kampo uses of *I. britannica*, experiment have been conducted on the survival rate of mice with acute hepatic failure provided by lipopolysaccharide (LPS) and propionibacterium acnes (PA) [66]. High dosage of lipolized supernatant fluid of *I. britannica* reduces the acute hepatic failure; thus increasing the survival rate. Using *Gardenia* fruit and *Caesalpinia* wood (already reported as hepatoprotective) as positive controls, supernatant of *I. britannica* was administered intraperitoneally at a dose of 4 mg/mouse/day to each group for 3 consecutive days before injecting LPS. Results showed that survival rate was 100% after 20 hours for *I. britannica* compared with 37.5% for the control group [67]. The study has also evaluated the production of spleen Th-cytokine and reported that IFNγ+ IL-4^-^ cells significantly increased in 24 h after LPS however, the group of mice receiving *I. britannica* showed insignificant change [67]. In conclusion, i*n vitro* tests recommend that *I. britannica* inhibit Th1 differentiation and induce Th2 differentiation by suppressing the production of macrophage IL-12 and promoting the production of IL-10. This in-turn restrains the immunology of hepatic injury by affecting the balance between Th1 and Th2 [67].

The bioassay guided isolation of taraxasteryl acetate from *I. britannica* (**13**) showed potent preventive activity against acute hepatic failure induced by PA and LPS in a dose-dependent manner that is in compliance with aforementioned study on extract. It was also observed that deacetylation and modification of olefinic bonds significantly decreased the anti-hepatitis activity of taraxasteryl acetate. Taraxasteryl acetate also inhibited the increment of plasma transaminase on acute hepatic failure induced by carbon tetrachloride (CCl4) or D-galactosamine. From a histological study, it appeared that degeneration and necrosis, which were observed in the liver induced by CCl4 mice, were not found in the liver cells from taraxasteryl acetate-treated mice. These results indicate that taraxasteryl acetate shows preventive effects on experimental hepatitis caused by either immunologically-induced injuries or hepatotoxic chemicals [38].

### 2.6. Enzyme Inhibition Activities

Many studies on animals and humans have reported the significant role of glutathione in antioxidant protection of lungs. In humans, glutathione concentrations in epithelial lining fluid are normally much higher than in blood. They have also been reported to be higher in epithelial lining fluid of smokers and patients with chronic obstructive pulmonary disease. The reported anti-oxidative flavonoids were tested against catalase, glutathione reductase and glutathione-peroxidase (GSH-px) [4]. Patuletin, nepetin, and axillarin showed significant decrease in glutathione induced by glutamate, which is associated with the oxidative stress by reducing the restrained glutathione. Patuletin resulted in 36.8, 14.6 and 11.9 µM consumed/min/mg protein compared with control 41.5, 18.1 and 13.2 µM consumed/min/mg proteins against Catalase, GSH-px and GSSG-R enzymes; Other compounds had lesser values compared to patuletin. This result showed non-simulative effect of flavonoids on the synthesis of glutathione. Furthermore, the oxidation of GSH induced by excess glutamate was counteracted by patuletin while the synthesis of GSH remained unaffected [4].

Glutathione is found higher in concentration in duodenum. The detoxifying capability of glutathione is related to the fact that glutathione regulates the action of glutathione-peroxidases and glutathione-transferases. A direct relationship has also been observed between glutathione concentration and mucosal damage or between glutathione-related enzymes present in various pathological conditions of gastrointestinal tract (from esophagus to rectum) [76].

Role of reported compounds (patuletin, nepetin, and axillarin) from *I. britannica* still needs to be studied under various physiological and pathological conditions in experimental animals and at a later stage, on man through clinical studies.

## 3. Conclusions

*I. britannica* alone or in combination with other plant species has widely been used for various diseases in TCM and Kampo Medicine. Indigenous uses of the plant created curiosity among scientists to isolate responsible bioactive constituents. Pursuing this, perspective metabolites were isolated from the plant. Most of the isolation work has been done on flowers and aerial parts of the plant, however, no information could be found on isolation of chemical constituents from roots. Mostly chloroform, ethyl acetate, or butanol fractions were used in isolation work. Limited work has however been done on the water fractions and saponins of the plant. Total syntheses of OABL and OODABL and some derivatives are available. In case of pharmacological investigations, results are obtained using animals’ cells by *in vitro* or *in vivo* experimentation. Several studies have elaborated the role of isolated compounds against various malfunctions at cellular and physiological levels. However, broader clinical trials on patients have yet to be performed.

Biosynthetic pathways of various phytochemicals, isolated (especially OABL and OODABL) from the plant, need to be elaborated so that a variety of physiological and pathological effects may be understood. Other sesquiterpenes, which are biologically screened and observed potent, are ignored for their possible significant effect in inflammation and carcinoma. Although isolated sesquiterpenes from *I. britannica* had been observed for anti-inflammatory and anticancer activities but antioxidant activities of purified sesquiterpenes were not found – which are widely reported as potent antioxidant too [68,69,70,71].

Metabolic pathways for important metabolites should be elaborated along with exploration of secondary metabolite synthesis in *I. britannica*. Studies should be planned so as to explore the allelopathic behavior of the plant. Studies explaining the ecological interactions with other species, wider distribution among various countries, production, trade and economic perspectives, and ethnobotany should be undertaken. Additional studies should be done to evaluate the genetic resources of the plant for variation in growth, morphology and yield-related characteristics which, in turn, can be used to identify high-yielding populations suitable for agronomical and plant breeding programs. Besides the ethnopharmacological recipes in TCM and Kampo Medicine, less or no work has been documented on the indigenous use of *I. britannica* by various marginal communities of world. Since, it has been reported to be an invasive weed species in various areas (including some US states) efforts are underway for its eradication. In doing so, conservational and medicinal importance of the plant has to be kept in mind.
